# A Case of Tæniæ Hydatigenæ, or Hydatids, Successfully Treated by the Use of Mercury

**Published:** 1789

**Authors:** James Lind

**Affiliations:** Physician at Windsor, Fellow of the Royal College of Physicians and of the Royal Society of Edinburgh


IV. *A Caſe of Tæniæ Hydatigenæ, or Hydatids, ſucceſsfully treated by the Uſe of Mercury. Communicated in a Letter to Dr.* Simmons, *F. R. S. by* James Lind, *M. D. F. R. S. Phyſician at Windſor, Fellow of the Royal College of Phyſicians and of the Royal Society of Edinburgh.*

ABOUT the end of October, 1786, I was ſent for to a married lady, about thirty years of age, who had been in a bad ſtate of health for ſome time, and then had a painful ſwelling at the pit of the ſtomach, and region of the liver. The diſorder had the appearance of hepatitis, and as if the liver were ſpeedily coming to ſuppuration: I therefore immediately began to give her quickſilver divided with mucilage of gum arabic, and made into pills, and to rub in mercurial ointment on the part affected, fected, in order to abate the inflammation and prevent ſuppuration *.

In about ten days the mercury began to affect her mouth, and at the ſame time ſhe voided an incredible quantity of the tæniæ hydatigenæ, or hydatides †, by ſtool and by vomiting. Her attendants reckoned ſhe paſſed to the number of a thouſand; there being as many as filled two large chamber pots. They were from the ſize of a ſmall pea to an inch and a half in diameter, and agreed exactly with the deſcription and figure of the lumbrices hydropici, given by Dr. Tyſon in the Philoſophical Tranſactions, No. 193. Many of theſe tæniæ hydatigenæ were deeply tinged with bile, ſome of which was alſo mixed with the gelatinous lymph with which they were filled, which ſhewed that they were alſo inhabitants of the biliary ducts as well

* *Vide* London Medical Journal, Vol. VIII. page 43.

† The tænia hydatigena, or hydatid, conſiſts of a double pellucid veſicle: the inner bag is of the ſhape of a ſmall bladder, the neck more opaque than the reſt of it, and is formed of a number of muſcular rings, with a ſmall orifice at the extremity. This vermicular part is endued with motion and other animal powers. The outer bag is but ſlightly attached to the inner one. The whole appears like a ſmall tranſparent bladder, which is about two thirds filled with lymph or ſerum.

as of the ſtomach and inteſtines, and that probably the liver alſo contained its ſhare of them: nor is it to be doubted but that they were the cauſe of the preſent malady in the liver, of the frequent pains in the ſtomach, and of the indigeſtion, with which the patient had been afflicted for above two years, during which period ſhe had, at intervals of about ſix months, paſſed ſome of theſe animals by ſtool. It ſeemed more than probable that the mercury, which I then gave her with a view to prevent the inflammation and ſuppuration of the liver, had rendered her juices poiſonous to the tæniæ hydatigenæ, and thus deſtroyed them.

Notwithſtanding the deſtruction of the tæniæ, the abſceſs was too far advanced to be diſperſed by the mercury, ſo that in a ſhort time it came to maturity, and pointed outwards. It was then agreed that it ſhould be opened; but in the night preceding the day appointed for the operation it burſt of itſelf near the pit of the ſtomach, and diſcharged a conſiderable quantity of fœtid, purulent matter at a ſmall orifice of about a quarter of an inch in diameter, which ſhe could not afterwards be prevailed upon to permit her ſurgeon to enlarge.

The

The difcharge continued for about two months, gradually decreafing in quantity, and at laft a gall-ftone of about the fize and fhape of a French bean paffed the orifice; after which the wound foon healed up, and in a few months after the patient perfectly recovered her health and ftrength, which fhe ftill continues to enjoy.

As moft of the encyfted dropfies which appear in different parts of the human body, befides a variety of other complaints, proceed from fuch collections of the tæniæ hydatigenæ, I hope the above cafe will prove acceptable to the readers of the London Medical Journal, as it may ferve to point out a method of removing fuch diforders.

*Windfor,*

January 1, 1789.

